# Confocal laser endomicroscopy in patients with acute respiratory failure

**DOI:** 10.1186/s40635-026-00909-1

**Published:** 2026-06-24

**Authors:** Kirsten A. Kalverda, Lizzy Wijmans, Lieuwe D. J. Bos, Mary R. Smit, Inge A. H. van de Berk, Daniel M. de Bruin, Paul Bloemen, Teodora Radonic, Marcus J. Schultz, Peter I. Bonta, Jouke T. Annema

**Affiliations:** 1 Department of Respiratory Medicine, Amsterdam UMC, University of Amsterdam, Amsterdam, The Netherlands; 2 Department of Intensive Care Medicine, Amsterdam, UMC, University of Amsterdam, Amsterdam, The Netherlands; 3 Department of Radiology, Amsterdam, UMC, University of Amsterdam, Amsterdam, The Netherlands; 4 Department of Biomedical Engineering and Physics, Amsterdam UMC, University of Amsterdam, Amsterdam, The Netherlands; 5 Department of Pathology, Amsterdam, UMC, University of Amsterdam, Amsterdam, The Netherlands; 6 Department of Anesthesia, General Intensive Care and Pain Management, Division of Cardiothoracic and Vascular Anesthesia & Critical Care Medicine, Medical University Wien, Vienna, Austria; 7 Department of Anesthesiology, Rescue– and Pain Medicine, Cantonal Hospital St. Gallen, HOCH Health Ostschweiz, St. Gallen, Switzerland; 8 Mahidol-Oxford Tropical Medicine Research Unit (MORU), Mahidol University, Bangkok, Thailand; 9 Nuffield Department of Medicine, Oxford University, Oxford, UK

**Keywords:** ARDS, Confocal laser endomicroscopy, Mechanical ventilation, Fibroproliferation

## Abstract

**Background:**

Survivors of the early exudative phase of acute respiratory distress syndrome (ARDS) may develop a fibroproliferative repair response and persistent microstructural remodeling associated with adverse outcomes. Conventional imaging, including chest computed tomography (CT), has limited biological specificity for early microscopic remodeling. Confocal laser endomicroscopy (CLE) enables real-time bronchoscopic imaging of the alveolar compartment with near-histologic resolution. We evaluated the feasibility and safety of bedside bronchoscopic CLE in invasively ventilated ICU patients with acute respiratory failure and explored whether in vivo CLE provides microscopic alveolar information complementary to chest CT.

**Methods:**

In this single-center observational pilot study, mechanically ventilated adult ICU patients with a clinical indication for bronchoalveolar lavage (BAL) underwent additional bedside bronchoscopic CLE. Primary endpoints were feasibility (≥ 1 interpretable alveolar video with visible septal architecture per procedure) and safety (CLE-related adverse events within 24 h). Secondary exploratory endpoints were dominant in vivo CLE patterns of alveolar filling (air, fluid, cells) and architecture (thin elastin fibers with hexagonal architecture; increased elastin with preserved architecture; increased elastin with distortion) and qualitative comparison with CT abnormalities in the imaged segments. As an exploratory additional analysis, ex vivo CLE was performed in a single autopsy case and compared with histopathology.

**Results:**

A total of 33 patients were included (median age 64 years; 61% male); 31/33 met Berlin ARDS criteria. Forty-one CLE procedures were performed, all yielding high-resolution alveolar imaging (procedural feasibility 100%). A total of 150 videos were acquired (mean acquisition time 46 s per video), with a mean of three bronchial segments imaged per procedure. No CLE-related adverse events occurred. Patterns that were recognized by CLE were: alveolar filling with air (*n* = 94, 63%), fluid (*n* = 18, 12%), cells (*n* = 38, 25%); alveolar architecture was described as thin elastin fibers with hexagonal architecture (*n* = 37; 25%), increased elastin fibers with preserved architecture (*n* = 80; 53%) or distorted architecture (*n* = 29; 19%). Architecture was undeterminable in 4 (3%) videos. CLE detected abnormalities in 6/7 CT-normal appearing segments and demonstrated architectural changes in 60/78 segments with ground-glass opacities. In the autopsy case, ex vivo CLE was concordant with histopathology: regions with increased and distorted CLE signal corresponded to thickened alveolar septa and fibrotic remodeling.

**Conclusion:**

Bedside bronchoscopic CLE is feasible and safe in mechanically ventilated ICU patients with ARDS. CLE provides complementary microscopic information on alveolar filling and architecture beyond chest CT, including features compatible with early structural remodeling.

## Background

Patients with severe acute respiratory failure requiring invasive ventilation are frequently classified as having acute respiratory distress syndrome (ARDS) [1]. ARDS, however, is a heterogeneous clinical syndrome. It is likely that this heterogeneity contributes to the repeated failure of interventions to improve clinical outcomes in unselected patients. Several approaches to identify more homogeneous ARDS subgroups have been proposed, including subphenotyping based on etiology, systemic inflammatory profiles, and radiological characteristics [2].

A direct histopathological approach to subphenotyping is rarely feasible, because surgical lung biopsy in these critically ill patients entails substantial procedural risk. Nevertheless, while diffuse alveolar damage (DAD) is classically linked to ARDS, autopsy data and limited open lung biopsy data indicate that DAD is present in only ~ 45% of patients fulfilling clinical ARDS criteria, underscoring the biological heterogeneity within the syndrome [3, 4]. Importantly, in selected cohorts undergoing surgical lung biopsy, histological information frequently leads to changes in clinical management, suggesting that distinct underlying pathological patterns are clinically meaningful [5]. Another clinically relevant aspect of heterogeneity is how lung injury evolves over time. Following an early exudative phase, some patients transition into fibroproliferative repair and, in a subset, ongoing microstructural remodeling, which has been associated with worse outcomes[6]. Chest CT and lung ultrasound are increasingly used in acute respiratory failure, but their ability to capture microscopic injury and repair is limited.

Confocal laser endomicroscopy (CLE) is a minimally invasive bronchoscopic imaging technique. CLE enables real-time imaging of the alveolar compartment with near histology resolution by visualization of the elastin fiber scaffold. In healthy volunteers the alveolar area is characterized by thin elastin fibers interconnected in hexagonal architecture with air-filled alveolar spaces [7]. An overview, including technical aspects and clinical implications, of this novel technique was published previously [8].

On a microscopic level, fibrotic remodeling is characterized by excessive extracellular matrix deposition, with collagen as a key component. However, increased and distorted elastin fibers within the alveolar network have also been described in fibrotic lung disease and in non-resolving fibroproliferative ARDS. Since structural remodeling can affect respiratory mechanics, bedside assessment of the elastin scaffold may provide clinically meaningful information beyond conventional imaging [9–11].

Given the marked biological heterogeneity underlying the clinical ARDS syndrome and the limited feasibility of obtaining histology in invasively ventilated ICU patients, bedside in vivo microscopy may provide complementary biological information to conventional imaging. We therefore conducted a single-center observational pilot study of bronchoscopic CLE in mechanically ventilated patients with acute respiratory failure.

The primary aim of this study was to assess procedural feasibility and safety. Secondary, exploratory aims were to describe the spectrum of in vivo CLE patterns of alveolar filling and architecture encountered at the bedside, and to explore whether these patterns show qualitative concordance with CT abnormalities in the CLE-imaged segments. We expected CLE to demonstrate distinct patterns of alveolar filling and architectural change, potentially beyond what CT can detect. We therefore explored qualitative concordance between CLE patterns and CT abnormalities within the same imaged segments. Data from this study have been presented in part as a conference abstract [12].

## Methods

### Study design and study cohort

This single-center observational pilot study was approved by the institutional review board of Amsterdam University Medical Center (NL61112.018.17) and subsequently extended under a follow-up protocol (NL76007.018.20). The extension enabled inclusion of patients with COVID-19 and allowed inclusion of an autopsy case for exploratory ex vivo measurements. Written informed consent was obtained from patients’ legal representatives prior to study procedures.

Eligible participants were adult ICU patients receiving invasive mechanical ventilation with a clinical indication for diagnostic bronchoscopy (including bronchoalveolar lavage). Patients were excluded if informed consent could not be obtained from legal representatives or if extracorporeal membrane oxygenation (ECMO) was in use.

Final clinical diagnosis of respiratory failure was established in a multidisciplinary meeting (including intensivists, pulmonologists, and a radiologist) using all available clinical data, excluding CLE images.

### Bronchoscopic CLE imaging

Chest CT scanning was performed prior to bronchoscopic CLE imaging and was used to guide segment selection. Although CLE imaging was primarily directed toward the most prominent CT abnormalities, a small number of CT-normal appearing segments were intentionally included, to explore whether CLE might reveal microscopic abnormalities not evident on Chest CT. CT scans were obtained as part of routine clinical care. Acquisition was attempted under inspiratory conditions (Siemens Somatom Force (Siemens Healthineers, Erlangen, Germany) Sn100 kV, reference mA 64, collimation 192 × 0.6, reconstruction thickness 1 mm, increment 1 mm, filter Bl57, lung window, Admire 3).

During diagnostic bronchoscopy, CLE imaging was performed using the Cellvizio^®^ 100 Series with the 1.4—mm AlveoFlex Confocal Miniprobe™ (Mauna Kea Technologies, Paris, France), providing real-time autofluorescence imaging (488—nm excitation, > 500—nm emission) with a field of view of 600 µm, lateral resolution up to 3.5 µm, and imaging depth up to 70 µm. CLE imaging was performed during ongoing mechanical ventilation; no breath-hold maneuvers were applied for image acquisition. Ventilator settings were not standardized for CLE, but remained at the clinically indicated settings determined by the treating team. The CLE probe was advanced through the bronchoscope working channel into the conducting airways, from here the CLE probe was gently pushed toward the periphery of the lung until the alveolar compartment was reached, defined by visualization of alveolar septae. Videos were acquired per imaged segment and stored for offline analysis.

### CT and CLE image assessment

All CLE videos were analyzed at the video level. Each video was linked to a CT-defined segment based on the bronchoscopic location where CLE imaging was performed.

#### CT evaluation

A chest radiologist assessed the dominant CT pattern in the imaged lung segments according to the Fleischner glossary of terms for thoracic imaging [13].

#### CLE assessment

Videos were scored for dominant patterns of alveolar filling and architecture. Alveolar architecture was categorized as: (1) thin elastin fibers with hexagonal architecture, (2) increased elastin fibers with preserved architecture, or (3) increased elastin fibers with distortion of normal alveolar architecture. Alveolar filling was classified as: air, fluid, or cells. Cellular alveolar filling’ was defined as intra-alveolar cellularity. As CLE does not permit definitive cell identification, this pattern was not subclassified by cell type.

Dominant alveolar filling and architecture patterns on CLE were assessed by two respiratory physicians experienced in CLE (KK and LW), who were blinded to CT findings.

#### Ex vivo* CLE–histology comparison*

In the event of a clinical autopsy, ex vivo CLE imaging was first performed on unprocessed excised lung tissue. The specimen was subsequently formalin-fixed and paraffin-embedded, sectioned, and the CLE probe was used to acquire images directly from the unstained tissue sections on glass slides, before hematoxylin and eosin (H&E) staining.

To enable 1:1 correlation, an overview “map” of the unstained section was generated using confocal laser scanning microscopy (Leica SP-8; excitation 488 nm, emission 505–554 nm; resolution 2 × 2 µm; scan area 17 × 24 mm). This was needed to relocate the exact CLE-imaged regions after staining. The overview scan was used solely for navigation and not for pattern classification. After H&E staining of the same section, histopathological findings were compared with the corresponding ex vivo CLE images to assess concordance, including features compatible with fibrotic remodeling.

### Study endpoints

The primary endpoints were feasibility and safety.

Feasibility was defined as the proportion of CLE procedures in which at least one interpretable alveolar video with septal architecture was successfully acquired.

Safety was defined as the occurrence of any adverse event judged to be related to CLE within 24 h after the procedure. Predefined events of interest included pneumothorax and clinically relevant airway bleeding or desaturation.

Secondary exploratory endpoints were [1] identification of dominant in vivo CLE patterns of alveolar filling and architecture and [2] qualitative comparison of CLE patterns with CT abnormalities in the imaged segments. An additional exploratory endpoint was ex vivo CLE–histopathology concordance in a single autopsy case.

### Sample size and statistical analysis

Given the exploratory pilot design, no formal sample size calculation was performed; a convenience sample of up to 35 inclusions was planned. Recruitment occurred when trained personnel and CLE equipment were available; apart from these operational constraints, no additional preselection criteria were applied.

Given the exploratory design, analyses were primarily descriptive. Continuous variables are reported as median (range) or mean (± SD), as appropriate; categorical variables as counts and percentages. CLE outcomes were analyzed at the video level and linked to the CT-defined segment in which imaging was performed. Cross-tabulations were used to summarize the distribution of dominant CLE alveolar filling and architectural patterns across dominant CT patterns. No formal hypothesis testing was planned and no adjustment for repeated procedures within patients was performed; results should therefore be interpreted as descriptive.

## Results

### Study cohort

Between October 11, 2017 and January 24, 2022, we enrolled 33 mechanically ventilated ICU patients with acute respiratory failure in a convenience sample. Inclusion depended on the on-site availability of trained staff and CLE equipment and recruitment was intermittently paused due to ICU operational constraints, including the early phase of the COVID-19 pandemic. Thereby, inclusion was primarily constrained by staff and equipment availability. The cohort had a median age of 64 years and was 61% male. All had bilateral opacities on chest CT. Thirty-one patients met the Berlin criteria for ARDS [14]; the remaining two had cardiogenic pulmonary edema (*n* = 1) and sputum plugging (*n* = 1). ARDS was predominantly pulmonary infection-related. Thirty-day ICU-mortality was 33% (11/33). Baseline characteristics are summarized in Table 1.

**Table 1:** Patient characteristics

	N = 33
Age *years* (median; range)	64; 25—78
Male (N; %)	20; 61%
Smoking status at the time of hospital admission	
Active smoker (N; %)	5; 15%
Non-smoker (N; %)	26; 84%--> 79%
Not reported (N; %)	2; 6%
Cause for acute respiratory failure	
ARDS (N; %)	31; 94%
Cardiogenic pulmonary edema (N; %)	1; 3%
Sputum plugging (N; %)	1; 3%
ARDS etiology (N = 31)	
Pneumonia (N; %)	25; 76%
COVID	18; 72%
Influenza	3; 9%
Other	4; 12%
Sepsis (N; %)	1; 3%
Severe trauma (N; %)	2; 6%
Mimic: panbronchiolitis (N; %)	1; 3%
Mimic: acute interstitial pneumonia (N; %)	1; 3%
Mimic: Aspergillus pneumonia (N; %)	1; 3%
ARDS severity at the time of CLE procedure (N = 41)	
Mild (N; %)	5; 12%
Moderate (N; %)	19; 46%
Severe (N; %)	15; 37%
Not applicable* (N; %)	2; 5%
30-day mortality (N; %)	11; 33%

At the time of imaging, patients had typically been ventilated for approximately 1 week, and CT was performed a median of 1 day before CLE (Table 2).

**Table 2:** CLE imaging procedures, CT patterns and CLE patterns

Number of patients, with at least 1 CLE imaging procedure (N)	33
Patients with 1 CLE procedure	27
Patients with 2 CLE procedures	4
Patients with 3 CLE procedures	2
Total number of in vivo CLE procedures (N)	41
Duration of invasive mechanical ventilation at CLE imaging *days* (median; range)	3 (0–24)
1st CLE procedure (n = 33)	5 (0–37)
2nd CLE procedure (n = 6)	12 (9–18)
3rd CLE procedure (n = 2)	25 (24–26)
Time between last CT scan and CLE imaging *days* (median; range)	1 (0–3)
Dominant CT pattern in successfully CLE imaged segments (*N* = 150)	
Normal (*N*; %)	7; 4.7%
Ground glass (*N*; %)	78; 52%
Consolidation (*N*; %)	28; 19%
Reticulation (*N*; %)	28; 19%
Tree in bud (*N*; %)	5; 3.3%
Atelectasis (*N*; %)	4; 2.7%
Dominant CLE imaged *alveolar filling pattern* (*N* = 150)	
Air (*N*; %)	94; 63%
Cells (*N*; %)	38; 25%
Fluid (*N*; %)	18; 12%
Dominant CLE imaged *alveolar architecture* (*N* = 150)	
Thin elastin fibers with hexagonal architecture (N; %)	37; 25%
Increased or thickened fibers with preserved architecture (N; %)	80; 53%
Increased fibers with distortion of architecture (N; %)	29; 19%
Undeterminable (N; %)	4; 2.6%

### Feasibility and safety

CLE imaging was feasible in all included patients and six patients underwent repeated imaging procedures (Table 2). One planned CLE procedure was cancelled due to oxygen desaturation during bronchoalveolar lavage (BAL) prior to CLE; this patient underwent three other successful CLE imaging procedures.

**Fig. 1:**
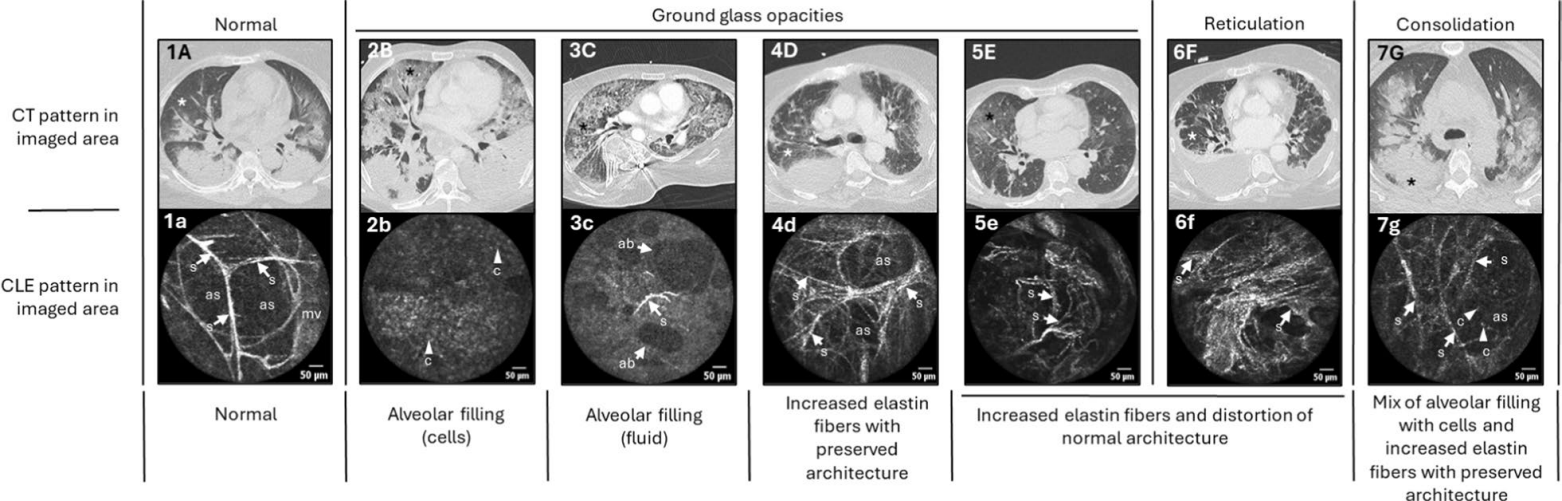
Thoracic CT scans showing the area of CLE imaging (asterisk, upper panel) and different corresponding CLE patterns (lower panel). 1A: CT scan showing bilateral consolidations, CLE imaging in normal area of lung parenchyma. 1a: corresponding CLE image showing elastin fibers which correspond with intact architecture of thin alveolar septae (white arrows) and rectangular airspaces (as), additionally an alveolar microvessel (mv) is partially visible. 2B: CT scan showing consolidation and ground glass opacities, in the imaged area ground glass is the dominant pattern. 2b: CLE showing an abundance of cellular alveolar filling, individual cells are indicated with white arrow head. 3C: CT scan showing a predominant pattern of bilateral ground glass opacities. 3c: CLE showing alveolar filling with fluid and air bubbles (white arrows). 4D:CT scan showing a predominant ground glass pattern. 4d: CLE showing increased elastin fibers (arrows), normal architecture with rectangular airspaces (as) are recognizable. 5E: CT scan showing bilateral ground glass opacities. 5e: CLE image with increased elastin fibers and distortion of normal architecture of alveolar septae (white arrows). 6F: CT scan showing a predominant pattern of reticulation in imaged segment. 6f: CLE image with increased elastin fibers and distortion of normal architecture of alveolar septae (white arrows). 7G: CT scan showing bilateral consolidations. 7 g: CLE image showing a combined pattern of some alveolar filling with cells (white arrowhead) and increased elastin fibers with normal recognizable architecture of alveolar septae (white arrows)

In total, 150 CLE videos were obtained (Fig. 1). Mean acquisition time per video was 46 s (range 11–222). A mean of three lung segments per patient were imaged, resulting in < 5 min additional bronchoscopy time.

One patient developed a right-sided pneumothorax within 24 h after CLE. After multidisciplinary review, this was considered to be unrelated to the CLE as the pneumothorax occurred directly after central venous line placement, a more likely explanation.

No clinically relevant bleeding occurred, and no procedure was terminated due to desaturation.

### CLE imaging assessment

Videos were scored for alveolar filling and architecture and linked to the corresponding CT pattern in the sampled segment (Table 3). In CT-normal segments (*n* = 7 videos), CLE showed fluid filling in four, cellular filling in two, and predominantly air-filled alveoli in one. Alveolar architecture was largely preserved; increased elastin signal was observed in two videos, and architecture could not be assessed in one due to extensive cellular filling (Fig. 1, Panel 1).

**Table 3:** CLE patterns stratified by CT pattern

		CLE pattern (N = 150)						
		Filling			Architecture			
		Air *N* = 94	Cells *N* = 38	Fluid *N* = 18	Thin elastin fibers preserved architecture *N* = 37	Increased elastin fibers preserved architecture *N* = 80	Increased elastin fibers distorted architecture *N* = 29	Undeterminable *N* = 4
CT pattern (*N* = 150)	Normal (*N* = 7)	1	2	4	4	2	0	1
	Ground glass (*N* = 78)	55	16	7	18	50	10	0
	Reticulation (*N* = 28)	21	5	2	0	11	17	0
	Consolidation (*N* = 28)	15	10	3	13	13	1	1
	Tree in bud (*N* = 5)	0	4	1	1	2	0	2
	Atelectasis (*N* = 4)	2	1	1	1	2	1	0

In segments with ground-glass opacity as the dominant CT pattern (*n* = 78), CLE showed predominantly air-filled alveoli in 55 (71%), cellular filling in 16 (21%), and fluid filling in 7 (9%) (Fig. 1, Panels 2–4). Among air-filled videos, 49/55 (89%) showed architectural abnormalities. The dominant architectural patterns were increased elastin fibers with preserved architecture (50/78; 64%) and increased elastin fibers with distortion (10/78; 13%) (Fig. 1, Panels 4–5). Normal architecture was observed in 18/78 (23%) videos; 12/18 (67%) of these still demonstrated alveolar filling.

In segments with dominant reticulation on CT (*n* = 28), all videos showed increased elastin signal. Increased elastin fibers with distortion was the dominant pattern in 17/28 (61%) videos (Fig. 1, Panel 6); in the remaining 11/28 (39%), increased elastin with relatively preserved architecture was dominant, although focal distortion was still present.

In consolidated segments (*n* = 28), CLE most often showed air-filled alveoli (15/28; 54%) or cellular filling (10/28; 36%). Architecture was classified as normal in 13/28 (46%) videos, of which 8/13 (62%) showed alveolar filling (cells *n* = 6; fluid n = 2). Five videos (5/28; 18%) demonstrated both predominantly air-filled alveoli and normal architecture. Increased elastin with preserved architecture was observed in 13/28 (46%) consolidation videos and distortion in 2/28 (7%) (Fig. 1, Panel 7). A full overview of CLE patterns stratified by CT pattern is provided in Table 3.

Of the videos showing cellular alveolar filling (38/150, 25%), 15 (39%) were obtained from patients who were active smokers at hospital admission, 21 (55%) from non-smokers, and 2 (5%) from patients whose smoking status was not reported (Table 1).

### Serial CLE imaging

Of the 150 videos, 126 were obtained during the first CLE procedure per patient, 13 during a second procedure, and 11 during a third. Alveolar filling decreased across follow-up procedures (first: 53/126 [42%]; second: 3/13 [23%]; third: 0/11 [0%]). Architectural abnormalities were common at all time points with increasing incidence over time (88/126 [70%], 11/13 [85%], and 10/11 [91%] for first, second, and third procedures, respectively). The proportion of videos showing increased elastin with distortion increased across procedures (20/126 [16%], 3/13 [23%], and 6/11 [55%]).

### Ex vivo CLE–histopathology comparison

**Fig. 2:**
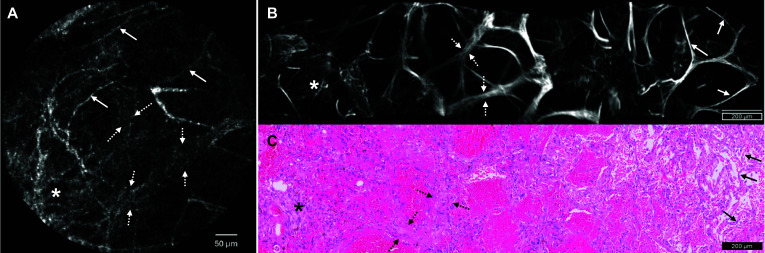
In vivo and ex vivo CLE imaging in COVID-19 ARDS with histologic correlation. Symbols are consistent across panels: solid arrows indicate thin alveolar septa; dotted arrows indicate septal thickening/increased elastin signal consistent with fibrotic remodeling; the asterisk marks an area with loss of recognizable alveolar architecture. **A** In vivo CLE acquired one day prior to death. **B** Ex vivo CLE mosaic from autopsy lung tissue. **C** Corresponding hematoxylin and eosin–stained section from the same specimen, also demonstrating intra-alveolar hemorrhage with airspaces filled by erythrocytes

**Fig. 3.**
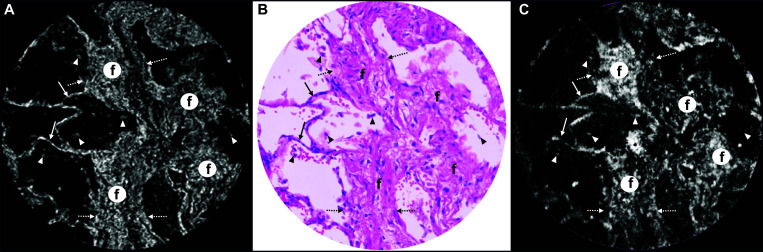
1:1 mapping of ex vivo confocal autofluorescence imaging to histology in autopsy lung tissue from fatal COVID-19 ARDS. Symbols are consistent across panels: arrowheads indicate individual intra-alveolar cells; solid arrows indicate thin alveolar septa; dotted arrows indicate septal thickening/increased elastin signal consistent with fibrotic remodeling (f). **A** Overview confocal laser scanning microscopy autofluorescence map of an unstained formalin-fixed paraffin-embedded tissue section (Leica SP-8; 488—nm excitation), used as a navigation map to relocate regions of interest for probe-based CLE. **C** Probe-based CLE image (Cellvizio**®** 100 Series) acquired from the mapped region on the same section, demonstrating increased signal along thickened septa in areas corresponding to fibrosis. **B** Subsequent H&E staining of the same section confirms fibrotic remodeling (increased collagen deposition and fibroblasts) in regions of increased autofluorescence/CLE signal. Together, the panels illustrate concordant spatial localization of fibrotic areas and the visualization of intra-alveolar cells

In one patient with COVID-19–associated ARDS, autopsy tissue enabled qualitative comparison of in vivo and ex vivo CLE with histopathology. In vivo CLE on day 12 after intubation (one day prior to death) showed prominently thickened elastin fibers with areas of preserved alveolar architecture and regions of distortion (Fig. 2A). Ex vivo CLE mosaics from the autopsy specimen demonstrated similar heterogeneity, with preserved areas alongside regions of increased signal and architectural distortion (Fig. 2B). Histopathology confirmed corresponding abnormalities, and areas of fibrotic remodeling on H&E aligned with regions of increased CLE signal, with fluorescence outlining thickened alveolar septa (Figs. 2C, 3). Although the in vivo and ex vivo images were obtained from the same specimen and likely represented overlapping regions, they did not provide an exact 1:1 spatial match. Using a slide-based mapping approach (Methods; Fig. 3A), ex vivo CLE acquired of an unstained tissue section could be matched 1:1 to H&E findings after staining of the same section (Fig. 3B–C). In these co-localized regions, areas showing fibrotic remodeling on histology corresponded to increased CLE signal outlining thickened alveolar septa. In addition to structural abnormalities, individual intra-alveolar cells could be identified (arrowheads; Fig. 3A and C).

## Discussion

This single-center observational pilot study demonstrates that confocal laser endomicroscopy (CLE) can be integrated into routine diagnostic bronchoscopy to provide real-time bedside imaging of the alveolar compartment in mechanically ventilated ICU patients with acute respiratory failure. Regarding our primary aims, procedural feasibility was 100% and CLE acquisition added less than five minutes to standard bronchoscopy, without CLE-related adverse events. As secondary, exploratory findings, CLE revealed distinct patterns of alveolar filling and architecture, and these patterns showed qualitative concordance with CT abnormalities in the imaged segments. Taken together, these data extend the limited ICU literature on CLE by linking in vivo alveolar micro-imaging to CT-defined segments and support the concept that bedside “alveolar microscopy” can complement conventional imaging in a biologically heterogeneous syndrome where histology is rarely available.

In the limited sampled CT-normal appearing segments, CLE revealed alveolar filling abnormalities in most cases, while in segments with ground-glass opacities it frequently showed architectural changes compatible with remodeling. Although these observations are exploratory and should be interpreted with caution, they are in line with prior reports that CT findings may be non-specific with respect to early fibroproliferative change in ARDS [15]. Although ground-glass opacities on CT are commonly attributed to edema, inflammation, or partial alveolar collapse, our CLE patterns suggest that microscopic architectural remodeling may already be present in a subset of these regions. Within CT-defined consolidations, a minority of sampled regions demonstrated preserved aeration and normal architecture on CLE, which may reflect atelectasis rather than parenchymal disease, although CLE sampling error cannot be excluded.

Beyond adding microscopic information to CT findings, CLE has practical advantages in the ICU. Lung ultrasound is an important bedside tool because it is non-invasive and radiation-free, but it remains limited to macroscopic patterns and cannot visualize alveolar microstructure. On the other hand, CLE requires bronchoscopy; however, in mechanically ventilated patients with acute respiratory failure, bronchoscopy with BAL is often clinically indicated, allowing CLE to be integrated into the same procedure with limited additional invasiveness and minimal prolongation. In this setting, CLE provides a direct, high-resolution view of the alveolar compartment, enabling assessment of septal architecture and intra-alveolar contents beyond the scope of ultrasound.

The present work primarily visualized the autofluorescent elastin scaffold, which is considered the dominant source of signal in CLE [16]. Although pulmonary fibrosis is classically characterized by collagen deposition, alterations of the elastin network accompany fibrotic remodeling and may explain why fibrotic change is detectable with CLE [9–11]. In the autopsy case, areas with increased or distorted CLE signal corresponded to thickened septa and fibrotic regions on histology. Ex vivo imaging of histology sections further suggested that collagen contributes to tissue autofluorescence, albeit likely to a lesser extent than elastin, supporting the concept that CLE can capture composite microstructural remodeling in vivo.

Cellular or fluid alveolar filling observed on CLE may be compatible with barrier disruption and distal airspace exudation in the exudative/inflammatory phase of ARDS, when neutrophils and macrophages are prominent drivers of injury. Previous in vivo CLE reports in ARDS and COVID-19 have described highly fluorescent floating structures, which were hypothesized to represent inflammatory cells containing protein–lipid components [17–19]. Interestingly, in patients with repeat procedures we observed less alveolar filling over time, alongside more architectural distortion and increased elastin signal. Although based on few follow-up procedures and susceptible to selection and survivor bias, this pattern is biologically plausible and may indicate a shift from inflammatory alveolar flooding toward fibroproliferative remodeling that may be difficult to appreciate on CT alone.

Regarding safety, no CLE-attributed complications were observed, which is in line with previous studies of CLE in patients with chronic lung disease [7, 20]. One patient developed a pneumothorax within 24 h; however, causality was considered unlikely in the context of concurrent procedures with a known pneumothorax risk. Nonetheless, given the small sample size and the vulnerability of this ICU population, definitive conclusions regarding safety require larger studies.

Several limitations merit consideration. Most of these limitations primarily affect the interpretation of the exploratory pattern and CT-comparison analyses rather than the primary feasibility and safety endpoints. This was an exploratory single-center study with a small convenience sample. Segment selection was CT-guided and therefore not random, enriching for abnormal regions; as a result, the study cannot reliably quantify specificity or false-positive rates in CT-normal lung. In addition, multiple videos and procedures per patient were analyzed without adjustment for within-patient clustering. CLE interpretation relied on expert consensus without formal inter-observer variability testing, and histopathological correlation was available only in a single autopsy case.

Importantly, these limitations should be interpreted in the context of the novelty of this work. To our knowledge, this is the first report of bedside CLE at this scale in mechanically ventilated patients. Clinically, bedside CLE may complement conventional imaging by adding microstructural and cellular information that otherwise would require high-risk bronchoscopic cryo- or surgical biopsy. As a future perspective CLE-based ARDS phenotyping has potential to add bedside microstructural information to support risk stratification and trial enrichment, but this needs prospective validation against outcomes. Given the pathological heterogeneity of clinical ARDS and the prognostic relevance of fibroproliferation [4, 21], improved bedside phenotyping could help identify patients with early structural remodeling, potentially identifying patients who could benefit from corticosteroid therapy, or even antifibrotic therapy [22, 23]. In addition, although the current study did not define or validate CLE patterns of alveolar collapse or overdistension, it did show that alveolar architecture can be visualized in vivo under dynamic breathing conditions. Future studies could therefore investigate whether specific CLE patterns may offer additional insight into microscopic differences between distinct lung areas in ARDS and if these patterns are associated with differences in recruitability, overstretch, or evolving ventilator-associated lung injury. Prospective studies should further evaluate reproducibility, define standardized descriptors, and test whether CLE-informed alveolar phenotyping improves clinical decision-making.

In conclusion, bedside bronchoscopic CLE is feasible and safe in mechanically ventilated ICU patients and provides a novel window into alveolar microstructure and filling patterns. Larger studies should evaluate whether CLE-derived descriptors can reproducibly phenotype acute respiratory failure and inform personalized management.

## Data Availability

Data will be available after publication for researchers with a valid research question, which will be evaluated by the study board. Researchers can address these requests by email to the corresponding author.

## Abbreviations

ARDS: Acute respiratory distress syndrome
BAL: Bronchoalveolar lavage
CLE: Confocal laser endomicroscopy
COVID-19: Coronavirus disease 2019
CT: Computed tomography
ECMO: Extracorporeal membrane oxygenation
H&E: Hematoxylin and eosin
ICU: Intensive care unit
SD: Standard deviation
SP-8: Confocal laser scanning microscope (Leica SP-8)
